# Association Between Subclinical Thyroid Dysfunction and Fracture Risk

**DOI:** 10.1001/jamanetworkopen.2022.40823

**Published:** 2022-11-08

**Authors:** Natalie R. Daya, Anna Fretz, Seth S. Martin, Pamela L. Lutsey, Justin B. Echouffo-Tcheugui, Elizabeth Selvin, Stephen P. Juraschek

**Affiliations:** 1 Department of Epidemiology, Johns Hopkins Bloomberg School of Public Health, Baltimore, Maryland; 2 Department of Medicine, University of California, San Francisco Medical Center, San Francisco; 3 Division of Cardiology, Department of Medicine, Ciccarone Center for the Prevention of Cardiovascular Disease, Johns Hopkins University School of Medicine, Baltimore, Maryland; 4 Division of Epidemiology and Community Health, University of Minnesota School of Public Health, Minneapolis; 5 Division of Endocrinology, Diabetes, and Metabolism, Department of Medicine, Johns Hopkins University School of Medicine, Baltimore, Maryland; 6 Department of Medicine, Beth Israel Deaconess Medical Center, Harvard Medical School, Boston, Massachusetts

## Abstract

**Importance:**

Clinical hyperthyroidism accelerates bone resorption without compensatory bone formation, reducing bone density and increasing the risk of fracture. The association between subclinical hyperthyroidism and fracture risk is less clear.

**Objective:**

To investigate the association of endogenous subclinical thyroid dysfunction and fracture risk, independent of clinical confounders.

**Design, Setting, and Participants:**

This cohort study included 10 946 participants from the Atherosclerosis Risk in Communities Study, an ongoing prospective cohort study of community-dwelling individuals conducted from 1987-1989 through December 31, 2019, in Washington County, Maryland; Forsyth County, North Carolina; Jackson, Mississippi; and the suburbs of Minneapolis, Minnesota. Participants were not taking thyroid medications and had no history of fractures.

**Exposures:**

Thyrotropin and free thyroxine levels were measured at visit 2 (1990-1992). Subclinical hyperthyroidism was defined as a thyrotropin level lower than 0.56 mIU/L, subclinical hypothyroidism as a thyrotropin level higher than 5.1 mIU/L, and euthyroidism as a thyrotropin level of 0.56 to 5.1 mIU/L, with normal free thyroxine levels from 0.85 to 1.4 ng/dL.

**Main Outcomes and Measures:**

Incident fracture was ascertained using hospitalization discharge codes through 2019 and linkage to inpatient and outpatient Medicare claims through 2018.

**Results:**

Of 10 946 participants (54.3% women; mean [SD] age, 57 [5.7] years), 93.0% had euthyroidism, 2.6% had subclinical hyperthyroidism, and 4.4% had subclinical hypothyroidism. During a median follow-up of 21 years (IQR, 13.0-27.3 years), there were 3556 incident fractures (167.1 per 10 000 person-years). The adjusted hazard ratios of fracture were 1.34 (95% CI, 1.09-1.65) for those with subclinical hyperthyroidism and 0.90 (95% CI, 0.77-1.05) for those with subclinical hypothyroidism compared with individuals with euthyroidism. Among those with normal free thyroxine levels, thyrotropin levels in the lower-than-normal range were significantly associated with higher fracture-related hospitalization risk; fracture risk was greater among individuals with thyrotropin concentrations below 0.56 mIU/L.

**Conclusions and Relevance:**

This community-based cohort study suggests that subclinical hyperthyroidism was an independent risk factor associated with fracture. The increased risk for fracture among individuals with a thyrotropin level lower than 0.56 mIU/L highlights a potential role for more aggressive screening and monitoring of patients with subclinical hyperthyroidism to prevent bone mineral disease.

## Key Points

**Question:**

What is the association between endogenous subclinical thyroid dysfunction and fracture risk?

**Findings:**

In this cohort study of 10 946 adults, 93% had euthyroidism, 2.6% had subclinical hyperthyroidism, and 4.4% had subclinical hypothyroidism. Those with subclinical hyperthyroidism had a 34% higher risk of fracture compared with individuals with euthyroidism.

**Meaning:**

This study suggests that subclinical hyperthyroidism was an independent risk factor associated with fracture, highlighting a potential role for more aggressive screening and monitoring of these patients to prevent bone mineral disease.

## Teaser

This cohort study uses data from the Atherosclerosis Risk in Communities Study to investigate the association between endogenous subclinical thyroid dysfunction and fracture risk, independent of clinical confounders.

## Introduction

Thyroid dysfunction is a well-established risk factor for bone-related outcomes, including fracture.^1,2,3^ Elevated levels of the thyroid hormones triiodothyronine and thyroxine, in the case of overt hyperthyroidism, accelerate bone resorption without compensatory bone formation, resulting in a net reduction of bone density.^4,5^ Suppressed levels of triiodothyronine and thyroxine, in the case of overt hypothyroidism, are associated with decreased bone resorption and either normal or slightly increased bone mass.^3,6,7,8^ It is suggested that the apparent increased risk of fractures for individuals with hypothyroidism could be explained by reduced bone remodeling and renewal, by confounding comorbid conditions, such as obesity or cardiovascular disease, or by neuromuscular complications that are associated with hypothyroidism and could lead to increased risk of bone fracture.^3^

Patients with overt thyroid dysfunction are usually symptomatic, readily diagnosed, and treated according to clinical guidelines: thyroid hormone replacement therapy in the case of overt hypothyroidism and antithyroid medications in the case of overt hyperthyroidism.^9,10,11^ However, there is no universal agreement on the indications for treating subclinical thyroid dysfunction.^12,13,14^

In the general adult population, subclinical hypothyroidism has an estimated prevalence of 12%, whereas subclinical hyperthyroidism is less common, with an estimated prevalence among US adults of approximately 5%.^15,16^ Moreover, subclinical thyroid dysfunction appears to persist as a chronic state for approximately 60% of individuals^17,18^ and is more prevalent among older adults.^19^ The existing literature on the association between subclinical thyroid dysfunction and bone-related outcomes has been conflicting.^20,21,22,23,24,25,26,27^ However, a meta-analysis of 13 studies found an independent positive association between subclinical hyperthyroidism and fracture risk.^28^ Many of the previous studies have been limited to cohorts of older and predominantly White patients, and they lack information on important confounders and the history of fracture. In addition, several studies included individuals using thyroid medications, thus limiting the literature on endogenous subclinical thyroid dysfunction.

We investigated the association of endogenous subclinical thyroid dysfunction with fracture risk, independent of clinical confounders, in a community-based cohort of middle-aged US adults who self-identified as Black or White. We assessed this association stratified by age, sex, and race.

## Methods

### Study Population

The Atherosclerosis Risk in Communities (ARIC) Study is a community-based prospective cohort of 15 792 participants sampled from 4 US communities: Washington County, Maryland; Forsyth County, North Carolina; Jackson, Mississippi; and the suburbs of Minneapolis, Minnesota. The study began in 1987 and recruited participants aged 45 to 64 years. Three additional follow-up visits were conducted every 3 years, and visits 5 to 8 occurred in 2011-2013, 2016-2017, 2018-2019, and 2020. Visit 9 is ongoing. The ARIC Study design and objective have been fully described previously.^29^ Institutional review boards at all study sites (University of Mississippi Medical Center [Jackson Field Center], Wake Forest Baptist Medical Center [Forsyth County Field Center], University of Minnesota [Minnesota Field Center], and Johns Hopkins University [Washington County Field Center]) approved the study, and written informed consent was obtained from all participants at each study visit. This study followed the reporting requirements of the Strengthening the Reporting of Observational Studies in Epidemiology (STROBE) reporting guideline statement.^30^

Thyroid function was first measured in stored blood samples collected during visit 2 in 1990-1992, which serves as the baseline for the present study. A total of 14 348 participants attended visit 2; of these, 13 500 had valid thyroid function measurements. We excluded individuals with a history of hospitalization for fracture at visit 2 (n = 121), those who self-reported race and ethnicity other than Black or White as well as Black individuals from Minnesota and Maryland due to the low counts in these catergories (n = 88), those missing follow-up information after baseline (n = 3), those missing information on the covariates of interest (n = 638), and those missing thyroid hormone measurements (n = 10). To limit our primary analyses to those with endogenous subclinical thyroid dysfunction, individuals taking any thyroid medication at visit 2 (n = 662) were excluded. Finally, 1032 participants were not classified in any of the 3 defined thyroid function categories (subclinical hypothyroidism, euthyroidism, or subclinical hyperthyroidism) because of abnormal free thyroxine levels, leaving a total of 10 946 participants who were included in the main analyses.

### Subclinical Thyroid Dysfunction

Subclinical thyroid dysfunction was characterized as having abnormal thyrotropin levels in the setting of normal free thyroxine levels, among those not taking thyroid medication at visit 2. Thyrotropin and free thyroxine levels were measured in 2012-2013 at the University of Minnesota from stored serum samples originally collected at visit 2 (1990-1992). Thyrotropin and free thyroxine levels were measured from serum samples on a Roche Elecsys 2010 Analyzer (Roche Diagnostics Corp) using a sandwich immunoassay method and competition immunoassay method, respectively. For the main analyses, we used ARIC-derived cut points to define subclinical thyroid dysfunction.^31^ Euthyroidism (reference group) was defined as a thyrotropin level of 0.56 to 5.1 mIU/L and a free thyroxine level of 0.85 to 1.4 ng/dL (to convert to picomoles per liter, multiply by 12.87). Subclinical hypothyroidism was defined as a thyrotropin level higher than 5.1 mIU/L, and subclinical hyperthyroidism was defined as a thyrotropin level lower than 0.56 mIU/L, both in the setting of a normal free thyroxine level ranging from 0.85 to 1.4 ng/dL.

### Fracture

The primary outcome was fracture, captured through the ARIC Study’s active hospitalization surveillance and linkage to Centers for Medicare & Medicaid Services (CMS) data. Individuals were eligible for CMS Medicare services when they turned 65 years of age or if they had a disability, end-stage kidney disease, or amyotrophic lateral sclerosis. Hospitalizations were identified from annual telephone contact with all study participants and through active surveillance of hospitalizations occurring in all study communities. Records of all inpatient hospital discharge codes are currently available for all ARIC Study participants through December 31, 2019. Inpatient and outpatient events are available from CMS data through December 31, 2018. Incident fracture was defined using *International Classification of Diseases, Ninth Revision* discharge codes of 733.1 to 733.19 (pathologic fractures), 733.93 to 733.98 (stress fracture), and 800 to 829 (fracture by injury) and the corresponding *International Statistical Classification of Diseases and Related Health Problems, Tenth Revision* codes for fractures that occurred after October 2015. The annual follow-up rate for the ARIC Study is greater than 80%. Participants who were lost to follow-up were administratively censored.

### Covariates

All covariates were measured at study baseline (visit 2). Body mass index was calculated from measured height and weight as weight in kilograms divided by height in meters squared. Diabetes was defined as self-reported physician diagnosis, current use of glucose-lowering medications, fasting blood glucose level of higher than or equal to 126 mg/dL (to convert to millimoles per liter, multiply by 0.0555), or random blood glucose level of higher than 200 mg/dL. High-density lipoprotein (HDL) cholesterol was assessed using standardized enzymatic methods.^32^ Hypertension-lowering medications were brought to the examination and recorded by trained staff. Smoking, alcohol consumption, and menopause status were self-reported. Physical activity was assessed at visit 1 by interview using a questionnaire developed by Baecke et al.^33^ An index for physical activity in sports was derived at visit 1, ranging from a score of 1 (low) to 5 (high).

### Statistical Analysis

The characteristics of the study population were examined by calculating the mean values for continuous variables and the proportions for categorical variables. We used Cox proportional hazards regression models to evaluate the association between baseline subclinical thyroid dysfunction and risk of incident fracture. Model 1 adjusted for age (years), sex (male or female), and race by study center (White individuals–Washington County, MD; White individuals–Minneapolis, MN; Black individuals–Jackson, MS; Black individuals–Forsyth County, NC; and White individuals–Forsyth County, NC). Model 2 was additionally adjusted for diabetes (yes or no), HDL cholesterol (milligrams per deciliter [to convert to millimoles per liter, multiply by 0.0259]), antihypertensive treatment (yes or no), body mass index, smoking status (current, former, or never), alcohol consumption (current, former, or never), physical activity score (score 1-5), menopause status (yes or no), and vitamin D level (nanograms per milliliter [to convert to nanomoles per liter, multiply by 2.496]). We performed likelihood ratio tests to test for interactions for race, sex, and age with thyroid function on the outcome of incident fracture. We calculated the mortality rate by thyroid dysfunction categories. We used the Fine-Gray method to incorporate the competing risk of death in the cumulative incidence function and generated subhazard ratios.^34^

We performed sensitivity analyses that (1) included participants who reported thyroid medication use at visit 2 to examine how fracture risk among those taking thyroid medication compared with individuals with euthyroidism and those with endogenous thyroid dysfunction; (2) redefined the outcome of fracture based on inpatient data only (ie, excluding outpatient fractures) to examine whether the association is strengthened by excluding the potentially less-severe outpatient fractures; (3) limited the outcome to nonpathologic fractures because it is plausible that pathologic fractures would have happened irrespective of thyroid hormone levels; (4) modeled diabetes, HDL cholesterol, antihypertensive treatment, body mass index, smoking status, alcohol consumption, and physical activity as time-varying covariates; and (5) modeled clinical thyroid dysfunction as a time-varying exposure. Clinical thyroid dysfunction was based on self-reported goiter or thyroid disease at visits 3 (1993-1995) or 4 (1996-1998); any thyroid medication use at visits 3, 4, 5 (2011-2013), 6 (2016-2017), or 7 (2018-2019); or overt hypothyroidism or hyperthyroidism at visit 5 based on available measurements of thyrotropin and free thyroxine. To more flexibly characterize the continuous association of thyrotropin level with fracture risk, we modeled log-transformed thyrotropin levels using restricted cubic and linear splines,^35^ among those with normal free thyroxine levels, with knots at the 5th, 27.5th, 50th, 72.5th, and 95th percentiles.

All statistical analyses were performed using Stata, version 17 (StataCorp LP). Statistical significance was defined a priori as a 2-sided *P* < .05.

## Results

At baseline, the 10 946 participants had a mean age of 57 (5.7) years, 24.0% were Black, 54.3% were female, 93.0% had euthyroidism, 2.6% had subclinical hyperthyroidism, and 4.4% had subclinical hypothyroidism (Table 1). Compared with individuals with euthyroidism and subclinical hypothyroidism, those with subclinical hyperthyroidism were more likely to be Black (euthyroidism, 24.0%; hypothyroidism, 10.7%; hyperthyroidism, 46.7%; *P* < .001), to have hypertension (euthyroidism, 31.8%; hypothyroidism, 26.4%; hyperthyroidism, 40.4%; *P* < .001), to have diabetes (euthyroidism, 14.3%; hypothyroidism, 11.6%; hyperthyroidism, 20.7%; *P* < .001), or to be a current smoker (euthyroidism, 22.1%; hypothyroidism, 11.8%; hyperthyroidism, 35.4%; *P* < .001). Persons with subclinical hypothyroidism were more likely to be older or female compared with those with euthyroidism and subclinical hyperthyroidism (mean [SD] age: hypothyroidism, 58.2 [5.7] years; euthyroidism, 56.7 [5.7] years; hyperthyroidism, 56.3 [5.7] years; *P* < .001; and female sex: hypothyroidism, 63.0%; euthyroidism, 53.8%; hyperthyroidism, 55.4%; *P* < .001).

**Table 1.** Characteristics According to Categories of Thyroid Dysfunction at Baseline ARIC Study Visit 2 (1990-1992) (N = 10 946)^a^

Characteristic	Mean (SD) value			*P* value^e^
	Subclinical hypothyroidism^b^	Euthyroidism^c^	Subclinical hyperthyroidism^d^	
Participants, No. (%)	484 (4.4)	10 177 (93.0)	285 (2.6)	NA
Thyrotropin level, mIU/L	7.4 (3.0)	2.0 (1.0)	0.4 (0.2)	<.001
Free thyroxine level, ng/dL	1.0 (0.1)	1.1 (0.1)	1.2 (0.1)	<.001
Age, y	58.2 (5.7)	56.7 (5.7)	56.3 (5.7)	<.001
Sex, No. (%)				
Male	179 (37.0)	4701 (46.2)	127 (44.6)	<.001
Female	305 (63.0)	5476 (53.8)	158 (55.4)	
Race, No. (%)				
Black	52 (10.7)	2441 (24.0)	133 (46.7)	<.001
White	432 (89.3)	7736 (76.0)	152 (53.3)	
BMI, No. (%)				
Obese (≥30)	128 (26.4)	2934 (28.8)	73 (25.6)	.32
Overweight (25 to <30)	188 (38.8)	4086 (40.1)	123 (43.2)	
Normal or underweight (<25)	168 (34.7)	3157 (31.0)	89 (31.2)	
Heart rate, beats/min	64.9 (10.0)	65.6 (10.1)	66.5 (10.9)	.10
Hypertension-lowering medication use, No. (%)	128 (26.4)	3239 (31.8)	115 (40.4)	<.001
Diabetes, No. (%)	56 (11.6)	1457 (14.3)	59 (20.7)	.002
HDL cholesterol, mg/dL	49.0 (16.4)	49.4 (16.7)	51.9 (18.4)	.04
Physical activity (sport index)	2.5 (0.8)	2.5 (0.8)	2.4 (0.8)	.05
Smoking, No. (%)				
Current	57 (11.8)	2247 (22.1)	101 (35.4)	<.001
Former	212 (43.8)	3865 (38.0)	101 (35.4)	.02
Never	215 (44.4)	4065 (39.9)	83 (29.1)	<.001
Drinking, No. (%)				
Current	296 (61.2)	5847 (57.5)	142 (49.8)	.009
Former	74 (15.3)	2081 (20.4)	87 (30.5)	<.001
Never	114 (23.6)	2249 (22.1)	56 (19.6)	.45
Menopause (among women; n = 5939)	250/305 (82.0)	4357/5476 (79.6)	132/158 (83.5)	.29
Vitamin D, ng/mL	23.6 (8.7)	22.8 (8.7)	21.9 (9.0)	.03

Abbreviations: ARIC, Atherosclerosis Risk in Communities; BMI, body mass index (calculated as weight in kilograms divided by height in meters squared); HDL, high-density lipoprotein; NA, not applicable.

SI conversion factors: To convert thyroxine to picomoles per liter, multiply by 12.87; HDL cholesterol to millimoles per liter, multiply by 0.0259; and vitamin D to nanomoles per liter, multiply by 2.496.

^a^ A total of 1032 participants were not classified in any thyroid function category owing to abnormal free thyroxine levels.

^b^ Subclinical hypothyroidism is defined as a thyrotropin level higher than 5.1 mIU/L and a normal free thyroxine level in the range of 0.85 to 1.4 ng/dL.

^c^ Euthyroidism is defined as a thyrotropin level in the range of 0.56 to 5.1 mIU/L and a normal free thyroxine level in the range of 0.85 to 1.4 ng/dL.

^d^ Subclinical hyperthyroidism is defined as a thyrotropin level lower than 0.56 mIU/L and a normal free thyroxine level in the range of 0.85 to 1.4 ng/dL.

^e^ The χ^2^ test was used to assess differences in categorical variables, and analysis of variance was used to test for differences in continuous variables.

During a median follow up of 21 years (IQR, 13.0-27.3 years), there were 3556 incident hospitalizations or outpatient events for fracture (167.1 fractures per 10 000 person-years). The rate of incident fracture per 10 000 person-years was 180.8 (95% CI, 155.5-210.3) among those with subclinical hypothyroidism, 165.8 (160.3-171.6) among those with euthyroidism, and 192.7 (157.8-235.4) among those with subclinical hyperthyroidism (Table 2). Of the 3556 incident fractures, 91.5% were fractures caused by injury, 12.0% were pathologic fractures, and 1.0% were stress fractures (eTable 1 in the Supplement). The most common locations for fractures were in the hip (14.1%) and spine 13.8%). The hazard ratio (HR) of fracture comparing those with subclinical hyperthyroidism with those with euthyroidism was 1.42 (95% CI, 1.16-1.74) after demographic adjustment (model 1) (Table 2). This association remained independent and robust even after adjustment for clinical and behavioral confounders (HR, 1.34 [95% CI, 1.09-1.65]) (model 2). There was no evidence for an association between subclinical hypothyroidism and fracture risk (HR, 0.90 [95% CI, 0.77-1.05]). These associations were generally consistent across fracture types including hip (model 1: HR for subclinical hyperthyroidism, 1.97 [95% CI, 1.00-3.88]), spine (model 1: HR for subclinical hyperthyroidism, 2.64 [95% CI, 1.29-5.42]), and nonspine (model 1: HR for subclinical hyperthyroidism, 1.52 [95% CI, 1.16-2.00]; eTable 2 in the Supplement). The mortality rate was highest among those with subclinical hyperthyroidism (27.3 per 1000 person-years [95% CI, 23.4-31.8 per 1000 person-years]; euthyroidism, 22.2 per 1000 person-years [95% CI, 21.6-22.8 per 1000 person-years]; hypothyroidism, 20.8 per 1000 person-years [95% CI, 18.3-23.7 per 1000 person-years]; eTable 3 in the Supplement). Accounting for the competing risk of death in the association between thyroid dysfunction and incident fracture did not substantially change our results (eTable 4 and eFigure in the Supplement).

**Table 2.** Adjusted Hazard Ratios for Fracture by Categories of Thyroid Dysfunction at Baseline^a^

Overall	Subclinical hypothyroidism	Euthyroidism	Subclinical hyperthyroidism
No./total No.	169/484	3291/10 177	96/285
IR (95% CI) per 10 000 person-years	180.8 (155.5-210.3)	165.8 (160.3-171.6)	192.7 (157.8-235.4)
Hazard ratio (95% CI)			
Unadjusted	1.09 (0.93-1.27)	1 [Reference]	1.21 (0.99-1.48)
Model 1^b^	0.87 (0.75-1.02)	1 [Reference]	1.42 (1.16-1.74)
Model 2^c^	0.90 (0.77-1.05)	1 [Reference]	1.34 (1.09-1.65)

Abbreviation: IR, incidence rate.

^a^ Fracture defined using Atherosclerosis Risk in Communities hospitalization and Centers for Medicare & Medicaid Services inpatient and outpatient data (3556 fractures).

^b^ Model 1: adjusted for age, sex, and race by center.

^c^ Model 2: adjusted for factors in model 1 plus diabetes, high-density lipoprotein cholesterol, antihypertensive treatment, heart rate, body mass index, smoking status, alcohol consumption, physical activity, menopause, and vitamin D level.

In analyses modeling thyrotropin level using a spline, lower-than-normal thyrotropin levels were significantly associated with increased fracture-related hospitalization risk, among those with a normal free thyroxine level. Fracture risk increased as thyrotropin levels decreased below 0.56 mIU/L (Figure; Table 2). Interactions by age, sex, and race were not statistically significant (eTable 5 in the Supplement).

**Figure.**
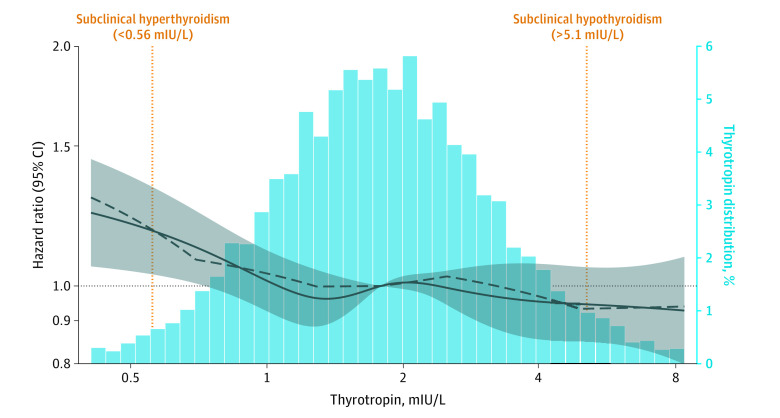
Adjusted Hazard Ratios for Baseline Thyrotropin Levels (Log Transformed) With Incident Fracture The adjusted hazard ratios are from Cox proportional hazards regression models. The thyrotropin levels were modeled using restricted cubic (solid curve) and piecewise linear splines (dashed curve) with knots at the 5th, 27.5th, 50th, 72.5th, and 95th percentiles. The shaded area indicates 95% CIs. The model is adjusted for age, sex, race by study center, diabetes, high-density lipoprotein cholesterol, antihypertensive treatment, heart rate, body mass index, smoking status, alcohol consumption, physical activity, menopause, and vitamin D level. The plot was truncated at the 1st and 99th percentiles of thyrotropin levels.

In sensitivity analyses that included individuals taking thyroid medications at visit 2 as a separate category, there was evidence of a slightly higher risk of fracture-related hospitalization among medication users compared with individuals with euthyroidism; however, it was not statistically significantly after adjusting for clinical and behavioral confounders (eTable 6 in the Supplement). Sensitivity analyses defining the outcome of fracture based only on inpatient data (ie, excluding outpatient fractures) resulted in a slightly stronger association with subclinical hyperthyroidism overall (HR, 1.54 [95% CI, 1.17-2.03] vs HR, 1.34 [95% CI, 1.09-1.65]) (eTable 7 in the Supplement). A sensitivity analysis excluding pathological fractures (n = 427) did not alter the association between thyroid function groups and fracture risk (eTable 8 in the Supplement). Modeling key covariates as time varying also did not appreciably alter the observed association (eTable 9 in the Supplement). Modeling clinical thyroid disease (medication use or self-reported thyroid disease) as a time-varying exposure attenuated the association between subclinical hyperthyroidism and fracture risk, although the association remained statistically significant (eTable 10 in the Supplement). We did not observe a significant association between incident clinical thyroid disease and fracture risk.

## Discussion

In this community-based cohort study of more than 10 000 participants, we found a statistically significant association between subclinical hyperthyroidism at baseline and fracture-related hospitalization but no evidence for an association between subclinical hypothyroidism and fracture-related hospitalization. In continuous analyses of thyrotropin levels, we found a significant risk of fracture-related hospitalization for suppressed levels of thyrotropin, even among those with normal free thyroxine levels. This study provides clinical insight into the management of subclinical thyroid dysfunction, for which there is currently insufficient evidence, to our knowledge.

Our observation of an association between subclinical hyperthyroidism and fracture risk is consistent with the literature. In a meta-analysis, Blum et al^28^ pooled individual participant data from 13 prospective cohort studies comprising 70 289 participants who had a median age of 64 years, were predominantly White, and had a median follow-up of 12 years. This study reported that subclinical hyperthyroidism was associated with a pooled relative risk of 1.28 (95% CI, 1.06-1.53) for all fractures, after adjustment for age and sex. In addition, no association was found between subclinical hypothyroidism and risk of fracture. Our study extends these findings to a younger, community-based cohort that included both Black and White participants, included extensive adjustment for potential confounders, and had a longer follow-up period (median follow-up of 21 years vs 12 years).

Our study found that suppressed thyrotropin levels were significantly associated with fracture risk among those with normal free thyroxine levels. Fracture-related hospitalization was highest for those with mildly to severely suppressed thyrotropin levels (<0.56 mIU/L). Further research is needed to confirm our findings and to better understand the biological mechanism by which subclinical thyroid dysfunction is associated with bone health. Those with mildly suppressed thyrotropin levels (0.1 mIU/L to <0.56 mIU/L) may benefit from being identified as a high-risk group through more aggressive screening. It also supports the existing recommendation, which is to treat all patients 65 years of age or older with subclinical hyperthyroidism when the thyrotropin level is persistently lower than 0.1 mIU/L for the prevention of bone mineral disease.^36^ Furthermore, it argues for a potential need for closer monitoring of those with a thyrotropin level between 0.1 and 0.56 mIU/L to prevent bone resorption.

The mechanisms linking subclinical thyroid dysfunction with fracture risk are not well understood. Prior studies demonstrate the presence of thyrotropin receptors in bone and inhibitory associations of thyrotropin level with bone resorption,^5,37^ which suggests a potential direct association of thyrotropin levels with bone metabolism. Although lacking strong epidemiologic evidence, this finding offers plausibility to the association between subclinical thyroid dysfunction and bone mass.

### Strengths and Limitations

This study has several strengths. The ARIC Study is a large, community-based prospective cohort of approximately 11 000 Black and White participants, with meticulous measurements of clinical risk factors and more than 30 years of follow-up for fracture. We were also able to link ARIC Study participants aged 65 or older with CMS claims data, providing more comprehensive surveillance for events. To our knowledge, this is the first study to demonstrate the association of subclinical thyroid dysfunction with fracture risk among Black and White individuals in a middle-aged community-based population.

There were also several limitations to our study that should be considered in the interpretation of our findings. Thyroid dysfunction was determined based on a single baseline measurement, which may have resulted in misclassification. To address this possibility, we conducted analyses with thyroid treatment and self-reported thyroid disease modeled as a time-varying variable, but these analyses were limited because we did not have comparable measurements of thyroid function tests with sufficient frequency to follow up over time. Nevertheless, prior studies have shown that thyroid function tests track reasonably well over time.^38,39^ The prevalence of subclinical thyroid dysfunction among the ARIC Study population was low because it is in the general population, resulting in limited power to detect interactions by important clinical subgroups. Given that ARIC Study participants are generally healthy and that most have euthyroidism, our results may differ from a clinical population with more prevalent and severe subclinical thyroid dysfunction. We had no assessment of bone mineral density, and we identified fractures using hospitalization discharge codes and CMS claims, which potentially have lower sensitivity than other definitions; however, this approach likely identified the cases of fracture that were most clinically important (high specificity). Although we adjusted for major confounders, residual confounding remains a possibility. Finally, the causes of hyperthyroidism and hypothyroidism are unknown and difficult to establish in our study owing to the absence of measurements of periodic thyroid antibodies and thyroid-stimulating immunoglobulins.

## Conclusions

This cohort study extends the evidence of an independent association between subclinical hyperthyroidism and fracture risk to a community-based, middle-aged cohort of White and Black individuals, identifying a higher-risk group that is relevant to guidelines focused on the screening and monitoring of patients with subclinical hyperthyroidism to prevent bone mineral disease. We also found that, among those with normal free thyroxine levels, those with suppressed thyrotropin levels have a significantly increased risk of fracture-related hospitalization, providing novel epidemiologic evidence that thyrotropin level may play an independent role in bone density and the risk of fracture. Longitudinal studies measuring thyroid function levels at more than 1 time point are needed to further examine the association of the trajectory of thyroid function with subsequent risk of fracture. Clinical trials are needed to elucidate the mechanisms by which subclinical thyroid dysfunction may be associated with fractures and other clinical outcomes.

## Notes

Supplement. **eTable 1.** Breakdown and *ICD-9* Codes for Different Types and Locations of Fractures (n=3,556) **eTable 2.** Adjusted Hazard Ratios for Fracture Types by Categories of Thyroid Dysfunction at Baseline **eTable 3.** Mortality Rate of Death by Categories of Thyroid Dysfunction at Baseline **eTable 4.** Adjusted Subhazard Ratios for Fracture, Incorporating the Competing Risk of Death **eTable 5.** Adjusted Hazard Ratios for Fracture by Categories of Thyroid Dysfunction at Baseline, Stratified by Race, Age, and Sex **eTable 6.** Adjusted Hazard Ratios for Fracture by Categories of Thyroid Dysfunction and Thyroid Medication Users at Baseline **eTable 7.** Adjusted Hazard Ratios for Inpatient Fracture by Categories of Thyroid Dysfunction at Baseline **eTable 8.** Adjusted Hazard Ratios for Fracture, Excluding Pathologic Fractures (n=427) **eTable 9.** Adjusted Hazard Ratios (95% CI) for Fracture by Categories of Thyroid Dysfunction at Baseline, Using Time-Varying Covariates **eTable 10.** Adjusted Hazard Ratios for Fracture by Categories of Subclinical Thyroid Dysfunction at Baseline and Incident Clinical Thyroid Dysfunction **eFigure.** Adjusted Cumulative Incidence of Fracture by Categories of Thyroid Dysfunction, Accounting for the Competing Risk of Death

## References

[1] Boelaert K, Franklyn JA. Thyroid hormone in health and disease. J Endocrinol. 2005;187(1):1-15. doi:10.1677/joe.1.06131 16214936

[2] Gorka J, Taylor-Gjevre RM, Arnason T. Metabolic and clinical consequences of hyperthyroidism on bone density. Int J Endocrinol. 2013;2013:638727. doi:10.1155/2013/638727 23970897 PMC3736466

[3] Vestergaard P, Mosekilde L. Fractures in patients with hyperthyroidism and hypothyroidism: a nationwide follow-up study in 16,249 patients. Thyroid. 2002;12(5):411-419. doi:10.1089/105072502760043503 12097203

[4] Uzzan B, Campos J, Cucherat M, Nony P, Boissel JP, Perret GY. Effects on bone mass of long term treatment with thyroid hormones: a meta-analysis. J Clin Endocrinol Metab. 1996;81(12):4278-4289. 8954028 10.1210/jcem.81.12.8954028

[5] Zaidi M, Davies TF, Zallone A, . Thyroid-stimulating hormone, thyroid hormones, and bone loss. Curr Osteoporos Rep. 2009;7(2):47-52. doi:10.1007/s11914-009-0009-0 19631028

[6] Vestergaard P, Rejnmark L, Mosekilde L. Influence of hyper- and hypothyroidism, and the effects of treatment with antithyroid drugs and levothyroxine on fracture risk. Calcif Tissue Int. 2005;77(3):139-144. doi:10.1007/s00223-005-0068-x 16151671

[7] Eriksen EF, Mosekilde L, Melsen F. Kinetics of trabecular bone resorption and formation in hypothyroidism: evidence for a positive balance per remodeling cycle. Bone. 1986;7(2):101-108. doi:10.1016/8756-3282(86)90681-2 3718785

[8] Mosekilde L, Eriksen EF, Charles P. Effects of thyroid hormones on bone and mineral metabolism. Endocrinol Metab Clin North Am. 1990;19(1):35-63. doi:10.1016/S0889-8529(18)30338-4 2192868

[9] Nordyke RA, Gilbert FI Jr, Harada AS. Graves’ disease: influence of age on clinical findings. Arch Intern Med. 1988;148(3):626-631. doi:10.1001/archinte.1988.00380030132023 3341864

[10] Singer PA, Cooper DS, Levy EG, . Treatment guidelines for patients with hyperthyroidism and hypothyroidism. JAMA. 1995;273(10):808-812. doi:10.1001/jama.1995.03520340064038 7532241

[11] Törring O, Tallstedt L, Wallin G, ; Thyroid Study Group. Graves’ hyperthyroidism: treatment with antithyroid drugs, surgery, or radioiodine—a prospective, randomized study. J Clin Endocrinol Metab. 1996;81(8):2986-2993. doi:10.1210/jcem.81.8.8768863 8768863

[12] Rugge JB, Bougatsos C, Chou R. Screening for and treatment of thyroid dysfunction: an evidence review for the U.S. Preventive Services Task Force. Report No. 15-05217-EF-1. Agency for Healthcare Research and Quality; 2014. Accessed October 20, 2022. https://www.ncbi.nlm.nih.gov/books/NBK285869/25927133

[13] Bahn RS, Burch HB, Cooper DS, . Hyperthyroidism and other causes of thyrotoxicosis: management guidelines of the American Thyroid Association and American Association of Clinical Endocrinologists. Endocr Pract. 2011;17(3):456-520. doi:10.4158/EP.17.3.456 21700562

[14] Cooper DS, Biondi B. Subclinical thyroid disease. Lancet. 2012;379(9821):1142-1154. doi:10.1016/S0140-6736(11)60276-6 22273398

[15] Hollowell JG, Staehling NW, Flanders WD, . Serum TSH, T(4), and thyroid antibodies in the United States population (1988 to 1994): National Health and Nutrition Examination Survey (NHANES III). J Clin Endocrinol Metab. 2002;87(2):489-499. doi:10.1210/jcem.87.2.8182 11836274

[16] Canaris GJ, Manowitz NR, Mayor G, Ridgway EC. The Colorado thyroid disease prevalence study. Arch Intern Med. 2000;160(4):526-534. doi:10.1001/archinte.160.4.526 10695693

[17] Das G, Ojewuyi TA, Baglioni P, Geen J, Premawardhana LD, Okosieme OE. Serum thyrotrophin at baseline predicts the natural course of subclinical hyperthyroidism. Clin Endocrinol (Oxf). 2012;77(1):146-151. doi:10.1111/j.1365-2265.2012.04345.x 22283624

[18] Huber G, Staub JJ, Meier C, . Prospective study of the spontaneous course of subclinical hypothyroidism: prognostic value of thyrotropin, thyroid reserve, and thyroid antibodies. J Clin Endocrinol Metab. 2002;87(7):3221-3226. doi:10.1210/jcem.87.7.8678 12107228

[19] McCahon D, Haque MS, Parle J, Hobbs FR, Roberts LM. Subclinical thyroid dysfunction symptoms in older adults: cross-sectional study in UK primary care. Br J Gen Pract. 2020;70(692):e208-e214. doi:10.3399/bjgp20X708065 PMC6960005 31932293

[20] Lee JS, Buzková P, Fink HA, . Subclinical thyroid dysfunction and incident hip fracture in older adults. Arch Intern Med. 2010;170(21):1876-1883. doi:10.1001/archinternmed.2010.424 21098345 PMC4122328

[21] Bauer DC, Ettinger B, Nevitt MC, Stone KL; Study of Osteoporotic Fractures Research Group. Risk for fracture in women with low serum levels of thyroid-stimulating hormone. Ann Intern Med. 2001;134(7):561-568. doi:10.7326/0003-4819-134-7-200104030-00009 12803168

[22] Mazziotti G, Porcelli T, Patelli I, Vescovi PP, Giustina A. Serum TSH values and risk of vertebral fractures in euthyroid post-menopausal women with low bone mineral density. Bone. 2010;46(3):747-751. doi:10.1016/j.bone.2009.10.031 19892039

[23] Svare A, Nilsen TI, Asvold BO, . Does thyroid function influence fracture risk? prospective data from the HUNT2 study, Norway. Eur J Endocrinol. 2013;169(6):845-852. doi:10.1530/EJE-13-0546 24031093

[24] Grimnes G, Emaus N, Joakimsen RM, Figenschau Y, Jorde R. The relationship between serum TSH and bone mineral density in men and postmenopausal women: the Tromsø study. Thyroid. 2008;18(11):1147-1155. doi:10.1089/thy.2008.0158 18925834

[25] Waring AC, Harrison S, Fink HA, ; Osteoporotic Fractures in Men (MrOS) Study. A prospective study of thyroid function, bone loss, and fractures in older men: the MrOS study. J Bone Miner Res. 2013;28(3):472-479. doi:10.1002/jbmr.1774 23018684 PMC4095773

[26] Garin MC, Arnold AM, Lee JS, Robbins J, Cappola AR. Subclinical thyroid dysfunction and hip fracture and bone mineral density in older adults: the Cardiovascular Health Study. J Clin Endocrinol Metab. 2014;99(8):2657-2664. doi:10.1210/jc.2014-1051 24878045 PMC4121038

[27] Földes J, Tarján G, Szathmari M, Varga F, Krasznai I, Horvath C. Bone mineral density in patients with endogenous subclinical hyperthyroidism: is this thyroid status a risk factor for osteoporosis? Clin Endocrinol (Oxf). 1993;39(5):521-527. doi:10.1111/j.1365-2265.1993.tb02403.x 8252739

[28] Blum MR, Bauer DC, Collet TH, ; Thyroid Studies Collaboration. Subclinical thyroid dysfunction and fracture risk: a meta-analysis. JAMA. 2015;313(20):2055-2065. doi:10.1001/jama.2015.5161 26010634 PMC4729304

[29] Wright JD, Folsom AR, Coresh J, . The ARIC (Atherosclerosis Risk In Communities) Study: JACC Focus Seminar 3/8. J Am Coll Cardiol. 2021;77(23):2939-2959. doi:10.1016/j.jacc.2021.04.035 PMC8667593 34112321

[30] von Elm E, Altman DG, Egger M, Pocock SJ, Gøtzsche PC, Vandenbroucke JP; STROBE Initiative. The Strengthening the Reporting of Observational Studies in Epidemiology (STROBE) statement: guidelines for reporting observational studies. Prev Med. 2007;45(4):247-251. doi:10.1016/j.ypmed.2007.08.012 17950122

[31] Schultheiss UT, Daya N, Grams ME, . Thyroid function, reduced kidney function and incident chronic kidney disease in a community-based population: the Atherosclerosis Risk in Communities study. Nephrol Dial Transplant. 2017;32(11):1874-1881. doi:10.1093/ndt/gfw301 27540046 PMC5837276

[32] National Heart Lung and Blood Institute. Atherosclerosis Risk in Communities (ARIC) Study: manual 8: lipid and lipoprotein determinations. Accessed January 9, 2022. https://sites.cscc.unc.edu/aric/sites/default/files/public/manuals/Lipid_and_Lipoprotein_Determinations.2_8.pdf

[33] Baecke JA, Burema J, Frijters JE. A short questionnaire for the measurement of habitual physical activity in epidemiological studies. Am J Clin Nutr. 1982;36(5):936-942. doi:10.1093/ajcn/36.5.936 7137077

[34] Fine JP, Gray RJ. A proportional hazards model for the subdistribution of a competing risk. J Am Stat Assoc. 1999;94(446):496-509. doi:10.1080/01621459.1999.10474144

[35] Harrell FE Jr, Lee KL, Pollock BG. Regression models in clinical studies: determining relationships between predictors and response. J Natl Cancer Inst. 1988;80(15):1198-1202. doi:10.1093/jnci/80.15.1198 3047407

[36] Donangelo I, Suh SY. Subclinical hyperthyroidism: when to consider treatment. Am Fam Physician. 2017;95(11):710-716. 28671443

[37] Mazziotti G, Sorvillo F, Piscopo M, . Recombinant human TSH modulates in vivo C-telopeptides of type-1 collagen and bone alkaline phosphatase, but not osteoprotegerin production in postmenopausal women monitored for differentiated thyroid carcinoma. J Bone Miner Res. 2005;20(3):480-486. doi:10.1359/JBMR.041126 15746993

[38] Sheehan MT. Biochemical testing of the thyroid: TSH is the best and, oftentimes, only test needed—a review for primary care. Clin Med Res. 2016;14(2):83-92. doi:10.3121/cmr.2016.1309 27231117 PMC5321289

[39] Karmisholt J, Andersen S, Laurberg P. Analytical goals for thyroid function tests when monitoring patients with untreated subclinical hypothyroidism. Scand J Clin Lab Invest. 2010;70(4):264-268. doi:10.3109/00365511003782778 20429701

