# Supplementary material for: Association Between Subclinical Thyroid Dysfunction and Fracture Risk
Source: JAMA Netw Open. 2022 Nov 8;5(11):e2240823. doi: 10.1001/jamanetworkopen.2022.40823 (PMC9644261; doi:10.1001/jamanetworkopen.2022.40823)
Supplement: Supplement. — eTable 1. Breakdown and ICD-9 Codes for Different Types and Locations of Fractures (n=3,556) eTable 2. Adjusted Hazard Ratios for Fracture Types by Categories of Thyroid Dysfunction at Baseline eTable 3. Mortality Rate of Death by Categories of Thyroid Dysfunction at Baseline eTable 4. Adjusted Subhazard Ratios for Fracture, Incorporating the Competing Risk of Death eTable 5. Adjusted Hazard Ratios for Fracture by Categories of Thyroid Dysfunction at Baseline, Stratified by Race, Age, and Sex eTable 6. Adjusted Hazard Ratios for Fracture by Categories of Thyroid Dysfunction and Thyroid Medication Users at Baseline eTable 7. Adjusted Hazard Ratios for Inpatient Fracture by Categories of Thyroid Dysfunction at Baseline eTable 8. Adjusted Hazard Ratios for Fracture, Excluding Pathologic Fractures (n=427) eTable 9. Adjusted Hazard Ratios (95% CI) for Fracture by Categories of Thyroid Dysfunction at Baseline, Using Time-Varying Covariates eTable 10. Adjusted Hazard Ratios for Fracture by Categories of Subclinical Thyroid Dysfunction at Baseline and Incident Clinical Thyroid Dysfunction eFigure. Adjusted Cumulative Incidence of Fracture by Categories of Thyroid Dysfunction, Accounting for the Competing Risk of Death [file jamanetwopen-e2240823-s001.pdf]

Supplementary Online Content

Daya NR, Fretz A, Martin SS, et al. Association between subclinical thyroid dysfunction and fracture risk. *JAMA Netw Open.* 2022;5(11):e2240823. doi:10.1001/jamanetworkopen.2022.40823

eTable 1. Breakdown and *ICD-9* Codes for Different Types and Locations of Fractures (n=3,556)
eTable 2. Adjusted Hazard Ratios for Fracture Types by Categories of Thyroid Dysfunction at Baseline
eTable 3. Mortality Rate of Death by Categories of Thyroid Dysfunction at Baseline
eTable 4. Adjusted Subhazard Ratios for Fracture, Incorporating the Competing Risk of Death
eTable 5. Adjusted Hazard Ratios for Fracture by Categories of Thyroid Dysfunction at Baseline, Stratified by Race, Age, and Sex
eTable 6. Adjusted Hazard Ratios for Fracture by Categories of Thyroid Dysfunction and Thyroid Medication Users at Baseline
eTable 7. Adjusted Hazard Ratios for Inpatient Fracture by Categories of Thyroid Dysfunction at Baseline
eTable 8. Adjusted Hazard Ratios for Fracture, Excluding Pathologic Fractures (n=427)
eTable 9. Adjusted Hazard Ratios (95% CI) for Fracture by Categories of Thyroid Dysfunction at Baseline, Using Time-Varying Covariates
eTable 10. Adjusted Hazard Ratios for Fracture by Categories of Subclinical Thyroid Dysfunction at Baseline and Incident Clinical Thyroid Dysfunction
eFigure. Adjusted Cumulative Incidence of Fracture by Categories of Thyroid Dysfunction, Accounting for the Competing Risk of Death

This supplementary material has been provided by the authors to give readers additional information about their work.

© 2022 Daya NR et al. *JAMA Network Open.*

eTable 1. Breakdown and *ICD-9* Codes for Different Types and Locations of Fractures (n=3,556)

**Type**	**ICD-9 code**	**n (% of fractures)**
**Pathological**	733.10-733.19	427 (12.0%)
**Stress**	733.93-733.98	36 (1.0%)
**Injury**	800-829	3,254 (91.5%)
**Location**	**ICD-9 code**	**n (% of fractures)**
**Hip**	820-820.9	502 (14.1%)
**Spine**	733.13; 805.2-805.5; 806.2-806.5	492 (13.8%)
**Non-spine**	Any fracture claims that do not contain the ICD-9 codes for spine	3,064 (86.2%)

© 2022 Daya NR et al. *JAMA Network Open.*

eTable 2. Adjusted Hazard Ratios for Fracture Types by Categories of Thyroid Dysfunction at Baseline

	Subclinical Hypothyroidism	Euthyroidism	Subclinical Hyperthyroidism
**N hip fractures**	24	465	13
**IR[a] (95% CI) per 10,000 person-years**	25.7 (17.2, 38.3)	23.4 (21.4, 25.7)	26.1 (15.2, 45.0)
**Model 1[b]**	1.25 (0.83, 1.89)	1 (Reference)	1.97 (1.00, 3.88)
**Model 2[c]**	1.28 (0.84, 1.93)	1 (Reference)	1.91 (0.96, 3.79)
**N spine fractures**	18	461	13
**IR[a] (95% CI) per 10,000 person-years**	19.3 (12.1, 30.6)	23.2 (21.2, 25.5)	26.1 (15.2, 45.0)
**Model 1[b]**	1.67 (1.04, 2.67)	1 (Reference)	2.64 (1.29, 5.42)
**Model 2[c]**	1.59 (0.99, 2.56)	1 (Reference)	2.18 (1.06, 4.49)
**N non-spine fractures**	151	2,830	83
**IR[a] (95% CI) per 10,000 person-years**	161.6 (137.7, 189.5)	142.6 (137.5, 148.0)	166.6 (134.4, 206.6)
**Model 1[b]**	1.09 (0.92, 1.28)	1 (Reference)	1.52 (1.16, 2.00)
**Model 2[c]**	1.06 (0.90, 1.25)	1 (Reference)	1.42 (1.08, 1.86)

[a] IR: Incidence rate

[b] Model 1: age, sex, race/center

[c] Model 2: Model 1 + diabetes, HDL cholesterol, antihypertensive treatment, heart rate, BMI, smoking status, alcohol consumption, physical activity, menopause, and Vitamin D level

© 2022 Daya NR et al. *JAMA Network Open.*

eTable 3. Mortality Rate of Death by Categories of Thyroid Dysfunction at Baseline

	n deaths/N	Mortality rate (95% CI) per 1,000 person-years
**Subclinical Hypothyroidism**	235/484	20.8 (18.3, 23.7)
**Euthyroidism**	5,193 /10,177	22.2 (21.6, 22.8)
**Subclinical Hyperthyroidism**	165/285	27.3 (23.4, 31.8)

© 2022 Daya NR et al. *JAMA Network Open*.

eTable 4. Adjusted[a] Subhazard Ratios for Fracture, Incorporating the Competing Risk of Death

	**sHR (95% CI)**
**Subclinical Hypothyroidism**	1.09 (0.93, 1.28)
**Euthyroidism**	1 (Reference)
**Subclinical Hyperthyroidism**	1.33 (1.02, 1.73)

[a]Adjusted for age, sex, race/center, diabetes, HDL cholesterol, antihypertensive treatment, heart rate, BMI, smoking status, alcohol consumption, physical activity, menopause, and Vitamin D level

© 2022 Daya NR et al. *JAMA Network Open.*

eTable 5. Adjusted Hazard Ratios for Fracture by Categories of Thyroid Dysfunction at Baseline, Stratified by Race, Age, and Sex

**Black individuals**	**Subclinical Hypothyroidism**	**Euthyroidism**	**Subclinical Hyperthyroidism**
**n/N**	11/52	546/2,441	45/133
IR (95% CI) per 10,000 person-years[a]	132.7 (73.5, 239.7)	117.7 (108.3, 128.0)	201.9 (150.7, 270.4)
Model 1[b]	0.87 (0.48, 1.58)	1 (Reference)	1.83 (1.35, 2.49)
Model 2[c]	0.91 (0.50, 1.66)	1 (Reference)	1.73 (1.27, 2.36)
**White individuals[d]**	**Subclinical Hypothyroidism**	**Euthyroidism**	**Subclinical Hyperthyroidism**
**n/N**	158/432	2,745/7,736	51/152
IR (95% CI) per 10,000 person-years[a]	186.8 (159.9, 218.3)	181.4 (174.8, 188.3)	185.4 (140.9, 243.9)
Model 1 [b]	0.86 (0.73, 1.01)	1 (Reference)	1.20 (0.91, 1.58)
Model 2 [c]	0.88 (0.75, 1.04)	1 (Reference)	1.12 (0.85, 1.48)
**60 years +**	**Subclinical Hypothyroidism**	**Euthyroidism**	**Subclinical Hyperthyroidism**
n/N	91/215	1,447/3,502	43/90
IR (95% CI) per 10,000 person-years[a]	261.9 (213.3, 321.7)	264.2 (250.9, 278.2)	379.9 (281.8, 512.3)
Model 1 [b]	0.88 (0.71, 1.09)	1 (Reference)	1.73 (1.27, 2.35)
Model 2 [c]	0.88 (0.71, 1.10)	1 (Reference)	1.63 (1.19, 2.21)
**Less than 60 years[e]**	**Subclinical Hypothyroidism**	**Euthyroidism**	**Subclinical Hyperthyroidism**
n/N	78/269	1,844/6,675	53/195
IR (95% CI) per 10,000 person-years[a]	132.8 (106.4, 165.9)	128.3 (122.6, 134.3)	137.7 (105.2, 180.3)
Model 1 [b]	0.86 (0.69, 1.09)	1 (Reference)	1.25 (0.95, 1.64)
Model 2 [c]	0.89 (0.71, 1.12)	1 (Reference)	1.18 (0.89, 1.55)
**Female**	**Subclinical Hypothyroidism**	**Euthyroidism**	**Subclinical Hyperthyroidism**
n/N	123/305	2,069/5,476	64/158
IR (95% CI) per 10,000 person-years[a]	206.0 (172.7, 245.9)	191.5 (183.4, 199.9)	223.2 (174.7, 285.2)
Model 1 [b]	0.89 (0.74, 1.07)	1 (Reference)	1.49 (1.16, 1.92)
Model 2 [c]	0.90 (0.75, 1.09)	1 (Reference)	1.38 (1.07, 1.77)
**Male[f]**	**Subclinical Hypothyroidism**	**Euthyroidism**	**Subclinical Hyperthyroidism**
n/N	46/179	1,222/4,701	32/127
IR (95% CI) per 10,000 person-years[a]	136.3 (102.1, 181.9)	135.2 (127.8, 143.0)	151.4 (107.1, 214.1)

© 2022 Daya NR et al. *JAMA Network Open.*

Model 1 [b]	0.81 (0.60, 1.09)	1 (Reference)	1.29 (0.91, 1.84)
Model 2 [c]	0.86 (0.64, 1.16)	1 (Reference)	1.26 (0.88, 1.79)

[a]IR: Incidence rate

[b] Model 1: age, race/center, sex

[c] Model 2: Model 1 + diabetes, HDL cholesterol, antihypertensive treatment, heart rate, BMI, smoking status, alcohol consumption, physical activity, menopause, and Vitamin D level

[d] p-value for interaction between thyroid category and race = 0.117

[e] p-value for interaction between thyroid category and age = 0.178

[f] p-value for interaction between thyroid category and sex = 0.773

© 2022 Daya NR et al. *JAMA Network Open*.

eTable 6. Adjusted Hazard Ratios for Fracture by Categories of Thyroid Dysfunction and Thyroid Medication Users at Baseline

	**Subclinical Hypothyroidism**	**Euthyroidism**	**Subclinical Hyperthyroidism**	**Thyroid Medication Users**
n/N	169/484	3,291/10,177	96/285	281/662
IR (95% CI) per 10,000 person-years[a]	182.0 (156.5, 211.6)	166.5 (160.9, 172.3)	192.8 (157.8, 235.4)	230.6 (205.2, 259.2)
Model 1[b]	0.88 (0.75, 1.02)	1 (Reference)	1.41 (1.15, 1.73)	1.13 (1.00, 1.28)
Model 2[c]	0.90 (0.77, 1.05)	1 (Reference)	1.33 (1.08, 1.63)	1.12 (0.99, 1.27)

[a]IR: Incidence rate

[b]Model 1: age, race/center, sex

[c]Model 2: Model 1 + diabetes, HDL cholesterol, antihypertensive treatment, heart rate, BMI, smoking status, alcohol consumption, physical activity, menopause, and Vitamin D level

© 2022 Daya NR et al. *JAMA Network Open.*

eTable 7. Adjusted Hazard Ratios for Inpatient Fracture[a] by Categories of Thyroid Dysfunction at Baseline

**Overall (n=10,965)**	**Subclinical Hypothyroidism**	**Euthyroidism**	**Subclinical Hyperthyroidism**
n/N	77/488	1,680/10,191	54/286
Model 1[b]	0.73 (0.58, 0.92)	1 (Reference)	1.66 (1.26, 2.18)
Model 2[c]	0.76 (0.60, 0.95)	1 (Reference)	1.54 (1.17, 2.03)

[a] Hospitalized Inpatient Fracture using ARIC Data and CMS Data, n=1,809 fractures
[b]Model 1: age, race/center, sex
[c]Model 2: Model 1 + diabetes, HDL cholesterol, antihypertensive treatment, heart rate, BMI, smoking status, alcohol consumption, physical activity, menopause, and Vitamin D level

© 2022 Daya NR et al. *JAMA Network Open.*

eTable 8. Adjusted Hazard Ratios for Fracture, Excluding Pathologic Fractures (n=427)

Overall (n=10,519)	Subclinical Hypothyroidism	Euthyroidism	Subclinical Hyperthyroidism
**n/N**	151/466	2,893/9,779	85/274
Model 1[a]	0.88 (0.74, 1.03)	1 (Reference)	1.43 (1.15, 1.77)
Model 2[b]	0.90 (0.76, 1.06)	1 (Reference)	1.36 (1.10, 1.69)

[a]Model 1: age, race/center, sex

[b]Model 2: Model 1 + diabetes, HDL cholesterol, antihypertensive treatment, heart rate, BMI, smoking status, alcohol consumption, physical activity, menopause, and Vitamin D level

© 2022 Daya NR et al. *JAMA Network Open.*

eTable 9. Adjusted Hazard Ratios (95% CI) for Fracture by Categories of Thyroid Dysfunction at Baseline, Using Time-Varying Covariates

**Overall (n=10,946)**	**Subclinical Hypothyroidism**	**Euthyroidism**	**Subclinical Hyperthyroidism**
**n/N**	169/484	3,291/10,177	96/285
Model 2[a]	0.90 (0.77, 1.05)	1 (Reference)	1.34 (1.09, 1.65)
Model 2b[b]	0.88 (0.75, 1.02)	1 (Reference)	1.37 (1.12, 1.68)

[a] Model 2: age, sex, race/center diabetes, HDL cholesterol, antihypertensive treatment, heart rate, BMI, smoking status, alcohol consumption, physical activity, menopause, and Vitamin D level

[a] Model 2b: age, sex, race/center, heart rate, menopause, and Vitamin D level; **time-varying:** diabetes, HDL cholesterol, antihypertensive treatment, BMI, smoking status, alcohol consumption, and physical activity

© 2022 Daya NR et al. *JAMA Network Open.*

eTable 10. Adjusted Hazard Ratios for Fracture by Categories of Subclinical Thyroid Dysfunction at Baseline and Incident Clinical Thyroid Dysfunction

	**Subclinical Hypothyroidism**	**Euthyroidism**	**Subclinical Hyperthyroidism**	**Incident clinical thyroid dysfunction[a]**
**n fractures**	118	3,074	79	285
**IR (95% CI) per 10,000 person-years[b]**	168.5 (140.7, 201.8)	162.9 (157.3, 168.8)	175.1 (140.5, 218.3)	226.6 (201.8, 254.5)
**Model 1[c]**	0.89 (0.74, 1.07)	1 (Reference)	1.36 (1.09, 1.70)	0.94 (0.83, 1.06)
**Model 2[d]**	0.92 (0.76, 1.11)	1 (Reference)	1.29 (1.03, 1.61)	0.93 (0.82, 1.05)

[a] Modeled as a time-varying exposure
[b] IR: Incidence rate
[c] Model 1: age, sex, race/center
[d] Model 2: Model 1 + diabetes, HDL cholesterol, antihypertensive treatment, heart rate, BMI, smoking status, alcohol consumption, physical activity, menopause, and Vitamin D level

© 2022 Daya NR et al. *JAMA Network Open.*

eFigure. Adjusted[a] Cumulative Incidence of Fracture by Categories of Thyroid Dysfunction, Accounting for the Competing Risk of Death

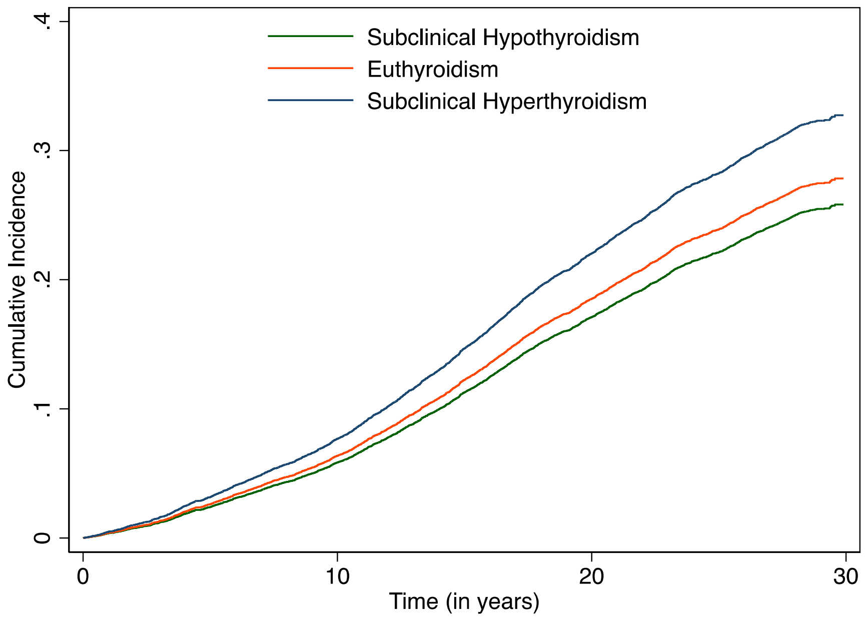

[a]Adjusted for age, sex, race/center, diabetes, HDL cholesterol, antihypertensive treatment, heart rate, BMI, smoking status, alcohol consumption, physical activity, menopause, and Vitamin D level

© 2022 Daya NR et al. *JAMA Network Open.*
